# Internal Administration of Belladonna in Cases of Severe Burn

**Published:** 1864-02

**Authors:** 


### INTERNAL ADMINISTRATION OF BELLADONNA IN CASES OF SEVERE BURN.

Experimental physiologists have recommended belladonna for use in the treatment of burns, in the belief that it diminishes that state of the nervous functions under which reflex inflammations are likely to be originated. They assert, on the other hand, that of all remedies opium is the one most powerful in increasing this peculiar state, and that it ought consequently to be avoided. In clinical practice, however, we believe that this opinion is wholly disregarded, and that opium is the form of anodyne most commonly resorted to in these cases. Yet it is generally suspected that the causes of death after burns are, in a majority of instances, connected with reflex inflammations, *e. g.*, ulcers of the intestine, pneumonia, &c. In a series of cases under Mr. Hutchinson's care in the London Hospital during the last six months, the belladonna treatment has been tried. In some remarks at the bedside of a patient the other day, Mr. Hutchinson stated that he considered the general results to have been fairly satisfactory. He adverted to the extreme difficulty of forming a trustworthy conclusion on such a matter, since these cases are, in their nature, never stationary, but always tend either to improvement or the reverse, and often with great rapidity. If, therefore, the remedy were commenced when the patient was very ill, it might chance to be just at the time when the improvement was about to set in; and if, on the other hand, the patient got worse, it might fairly be alleged that the remedy was used too late. If, on the other hand, we should give it in cases in which, as yet, no serious symptoms had appeared, we might again be much led astray, since a great majority of burn cases do well without any special plan of medication. Mr. Hutchinson stated that the cases in which the remedy had seemed to be most useful, were those of children in whom general febrile symptoms, attended with restlessness, loss of appetite, &c., had set in without any local complications. In several of these, there could be no mistake that the feverish state had passed away quickly and very satisfactorily under the use of belladonna. In no cases had he witnessed any ill results. If the burn itself was very painful, and the patient unable to get sleep on account of the pain, then the belladonna seemed comparatively inefficacious to procure ease, and morphia was far more efficient. As a rule, no opium had been given to the cases treated by belladonna; but in a few, and those chiefly in adults, it had been found requisite to give an occasional night dose. Possibly more benefit might have been obtained had the administration of the belladonna been pushed to larger doses. The usual dose given had been a third of a grain three times a day. In speaking of the less frequent results of burns, Mr. Hutchinson mentioned a recent case in which acute inflammation of one hip-joint, followed rapidly by dislocation, had occurred in a child who had been severely burnt on the arm and chest. He was in doubt whether to regard it as a reflex inflammation, or as a consequence of pyæmia.—*Med. Times and Gazette, Jan.* 2, 1864.

